# Supplementary material for: Efficient Quantification of Lipid Packing Defect Sensing by Amphipathic Peptides: Comparing Martini 2 and 3 with CHARMM36
Source: J Chem Theory Comput. 2022 Jun 16;18(7):4503–14. doi: 10.1021/acs.jctc.2c00222 (PMC9281404; doi:10.1021/acs.jctc.2c00222)
Supplement: Supplementary file 1 — ct2c00222_si_001.pdf [file ct2c00222_si_001.pdf]

Supporting Information:

Efficient quantification of lipid packing defect sensing by amphipathic peptides; comparing Martini 2 & 3 with CHARMM36

Niek van Hilten,[†] Kai Steffen Stroh,[‡,¶] and Herre Jelger Risselada[*,†,‡,¶]

†*Leiden Institute of Chemistry, Leiden University, Leiden, The Netherlands*

‡*Department of Physics, Technical University Dortmund, Dortmund, Germany*

¶*Institute for Theoretical Physics, Georg-August-University Göttingen, Göttingen Germany*

E-mail: jelger.risselada@tu-dortmund.de

1 Derivation of the relation between relative strain and vesicle diameter

Membrane curvature can be modeled by the stretching and compression of two uncoupled elastic sheets. Consequently, when the outer monolayer is bent, the area of the neutral plane within the outer leaflet remains constant, whereas the region above this plane is stretched, and the region below this plane is compressed. Now, we define d as the distance from the head groups to the neutral plane of the outer monolayer, i.e, approximately half the monolayer thickness, and R as the distance from the vesicle center to this neutral plane (Fig. SI1). The relative strain ϵ (area increase) between the leaflet area at the position of the head groups $R+d$ and the neutral plane R can be derived via the different spherical areas:

$$\epsilon = \frac{A - A_0}{A_0} = \frac{4\pi(R+d)^2 - 4\pi R^2}{4\pi R^2} = \frac{R^2 + 2dR + d^2}{R^2} - 1 = \frac{d^2}{R^2} + \frac{2d}{R} \tag{1}$$

Which can be rearranged to find the effective vesicle radius R as a function of ϵ:

$$R = \frac{d(1+\sqrt{1+\epsilon})}{\epsilon} \tag{2}$$

This expression of R is equivalent to the result obtained by others,[1] with the difference that we base our approximation on the neutral plane of the outer monolayer rather than the bilayer when bending two uncoupled elastic sheets. Now, if we plug in the numbers for our system ($\epsilon = 0.165$ and $d \simeq 1$ nm), we find that this corresponds to an effective vesicle radius of ≈ 12.5 nm and, consequently, a diameter in the range of 25 nm. Though it should be noted that such a simple estimation remains rather approximate since it is based on the assumption of a uniform lateral stretching modulus.

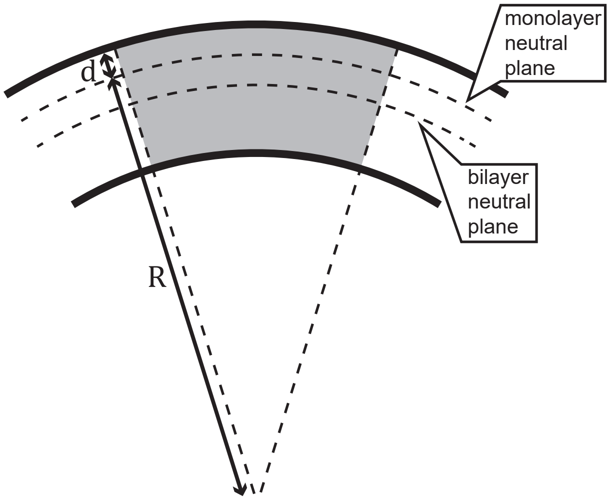

Figure SI1: Schematic depiction of a bent membrane, with the relevant distances R and d.

2 Peptide sequences and properties

Table SI1: Sequences and physicochemical properties for the six peptides studied in this work. Mutated amino acids (in HCV NH and M2 MH/NH) with respect to the original peptides (HCV AH and M2 AH, respectively) are highlighted in gray. Hydrophobicities ($< H >$) and hydrophobic moments ($< \mu_H >$) were calculated for the full sequences by HeliQuest[2] (default settings).

Peptide	Sequence	Length	Charge	$< H >$	$< \mu_H >$
ALPS	DDFLNSAMSSLYSGWSSFTTGASKFASAAKEGATK	35	0	0.279	0.365
HCV AH	SGSWLRDVWDWICTVLTDFKTWLQSKL	27	0	0.686	0.465
HCV NH	SGSWLRDDWDWECTVLTDDKTWLQSKL	27	-3	0.221	0.427
M2 AH	RLFFKCIYRFFEHGLKRG	18	+4	0.519	0.474
M2 MH	RKFFKKIYRFFRKLLKRL	18	+9	0.335	0.874
M2 NH	RLAAKCAARFAEHGLKRG	18	+4	0.153	0.263

3 The relation between sensing free energies in constant area and constant tension ensembles

The stretching free energy at constant area ($\Delta F|_A$) can be extended to the free energy at constant tension ($\Delta G|_\sigma$) by using the Legendre transformation:

$$dG|_\sigma = \Big(\frac{\partial G}{\partial A}\Big)\Big|_A dA + \Big(\frac{\partial G}{\partial \sigma}\Big)\Big|_\sigma d\sigma = \sigma dA\big|_A + \Delta A d\sigma\big|_\sigma \tag{3}$$

Note that we only use ΔF to emphasize that the free energies are obtained within the constant area ensemble. However, both ensembles are performed at a constant pressure of $p_z = 1$ bar. When we integrate this between the tensionless state (labeled 0) and the high tension state (labeled 1), we find

$$\Delta G|_\sigma = \int_{A_0}^{A_1} \sigma dA\big|_A + \int_{\sigma_0}^{\sigma_1} \Delta A d\sigma\big|_\sigma = \underbrace{\frac{A_1 - A_0}{2}(\sigma_1 + \sigma_0)\Big|_A}_{\Delta F|_A} + \underbrace{(A_1' - A_1)\sigma_1\Big|_{\sigma_1}}_{\Delta G|_{\sigma_1}} - \underbrace{(A_0' - A_0)\sigma_0\Big|_{\sigma_0}}_{\Delta G|_{\sigma_0}} \tag{4}$$

for the system without a peptide adhered to the membrane surface. The first term ($\Delta F|_A$) is identical to the definition of eq. 6 in the main text. The two correction terms represent the constant tension contributions to the stretching free energies at high tension ($\Delta G|_{\sigma_1}$) and tensionless conditions ($\Delta G|_{\sigma_0}$). Similarly, for the system with an adhered peptide to the membrane surface we find:

$$\Delta G'|_{\sigma'} = \int_{A_0'}^{A_1'} \sigma' dA\big|_{A'} + \int_{\sigma_0'}^{\sigma_1'} \Delta A' d\sigma\big|_{\sigma'} = \underbrace{\frac{A_1' - A_0'}{2}(\sigma_1' + \sigma_0')\Big|_{A'}}_{\Delta F'|_{A'}} + \underbrace{(A_1' - A_1)\sigma_1'\Big|_{\sigma_1'}}_{\Delta G'|_{\sigma_1'}} - \underbrace{(A_0' - A_0)\sigma_0'\Big|_{\sigma_0'}}_{\Delta G'|_{\sigma_0'}} \tag{5}$$

As an example, we performed additional simulations for the ALPS peptide with surface-tension coupling to the surface tensions we found in the constant area runs for the four conditions (σ_0, σ_0', σ_1 , and σ_1'). This showed that the correction terms for the tensionless

membrane systems, $\Delta G|_{\sigma_0}$ and $\Delta G'|_{\sigma'_0}$, are indeed negligibly small ($0.12{\cdot}10^{-3}$ and $-6.29{\cdot}10^{-3}$ kJ mol^{-1}, respectively), because σ_0 and σ'_0 are – per definition – close to zero in the tensionless state. Also, the correction terms for the tensed states, $\Delta G|_{\sigma_1}$ and $\Delta G'|_{\sigma'_1}$, are nearly identical (0.697 and 0.650 kJ mol^{-1}, respectively), such that they almost entirely cancel out when calculating the sensing free energy ($\Delta\Delta G_{sensing}\big|_{\sigma,A} = \Delta G'|_{\sigma'} - \Delta G|_{\sigma}$).

Taken together, we find a corrected sensing free energy $\Delta\Delta G_{sensing}\big|_{\sigma,A}$ of -12.077 kJ mol^{-1} for ALPS, which is merely 0.040 kJ mol^{-1} smaller than the value we find when only simulating the systems at constant area ($\Delta\Delta F_{sensing}\big|_{A} = -12.037$ kJ mol^{-1}, see main text). From this, we conclude that the constant area simulations of the two end-states yield sufficiently reliably free energy values and do not require additional simulations of the constant tension ensembles.

4 CHAOS parameters compared to the area increase upon peptide adhesion

Table SI2: Calculated CHAOS parameters (eq. 8, Fig. 3D in main text) compared to the induced area upon peptide-membrane adhesion in an NpT ensemble (tensionless conditions, average and standard error over 1 μs of simulation).

Peptide	CHAOS parameter (nm^2)	Area induction in NpT (nm^2)
ALPS	0.611 ± 0.029	0.513 ± 0.015
HCV AH	0.691 ± 0.032	0.619 ± 0.015
HCV NH	0.034 ± 0.007	0.026 ± 0.017
M2 AH	0.408 ± 0.022	0.329 ± 0.020
M2 MH	0.566 ± 0.028	0.450 ± 0.013
M2 NH	0.202 ± 0.012	0.112 ± 0.031

5 $\frac{\partial V}{\partial \lambda}$ -profiles for thermodynamic integration

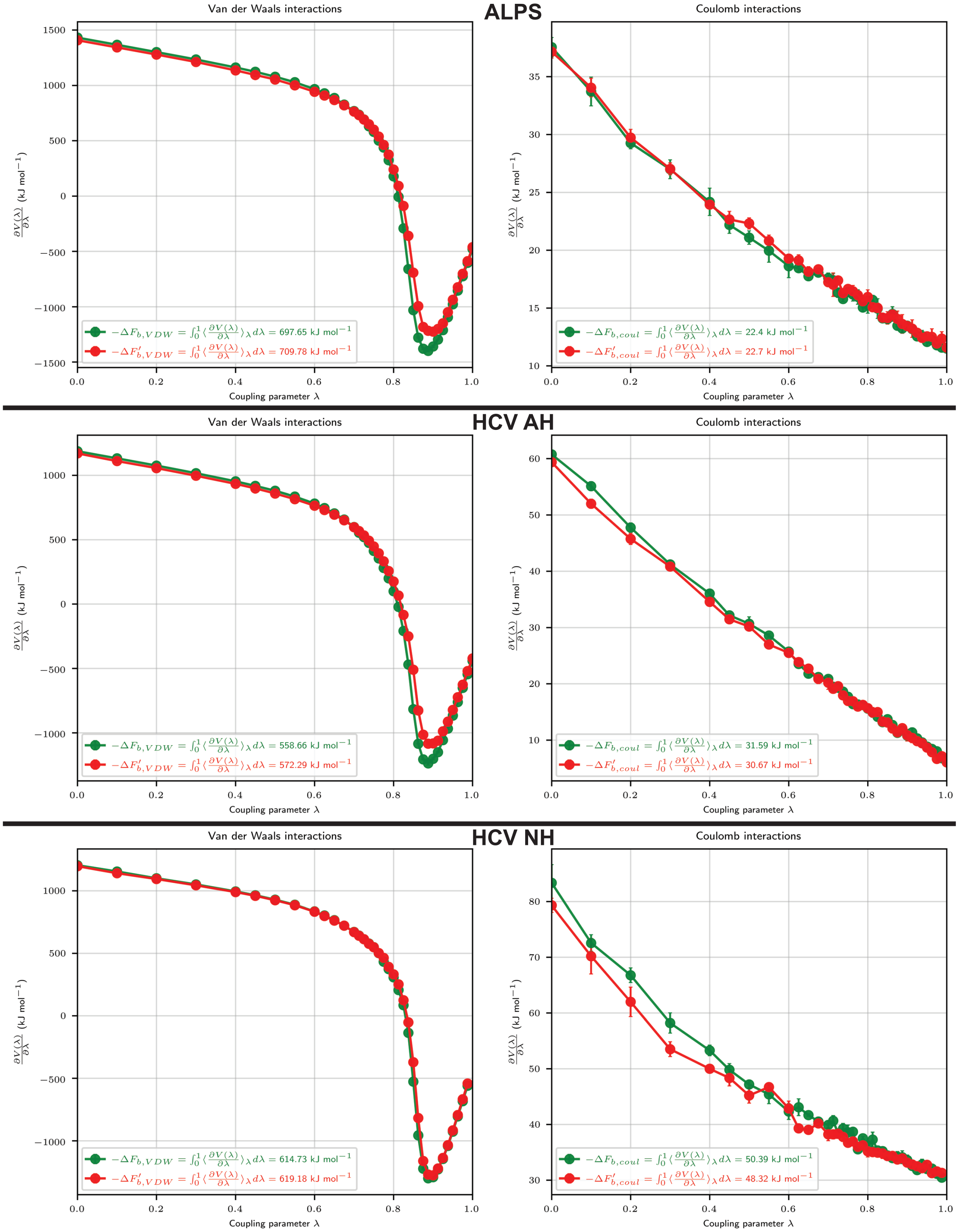

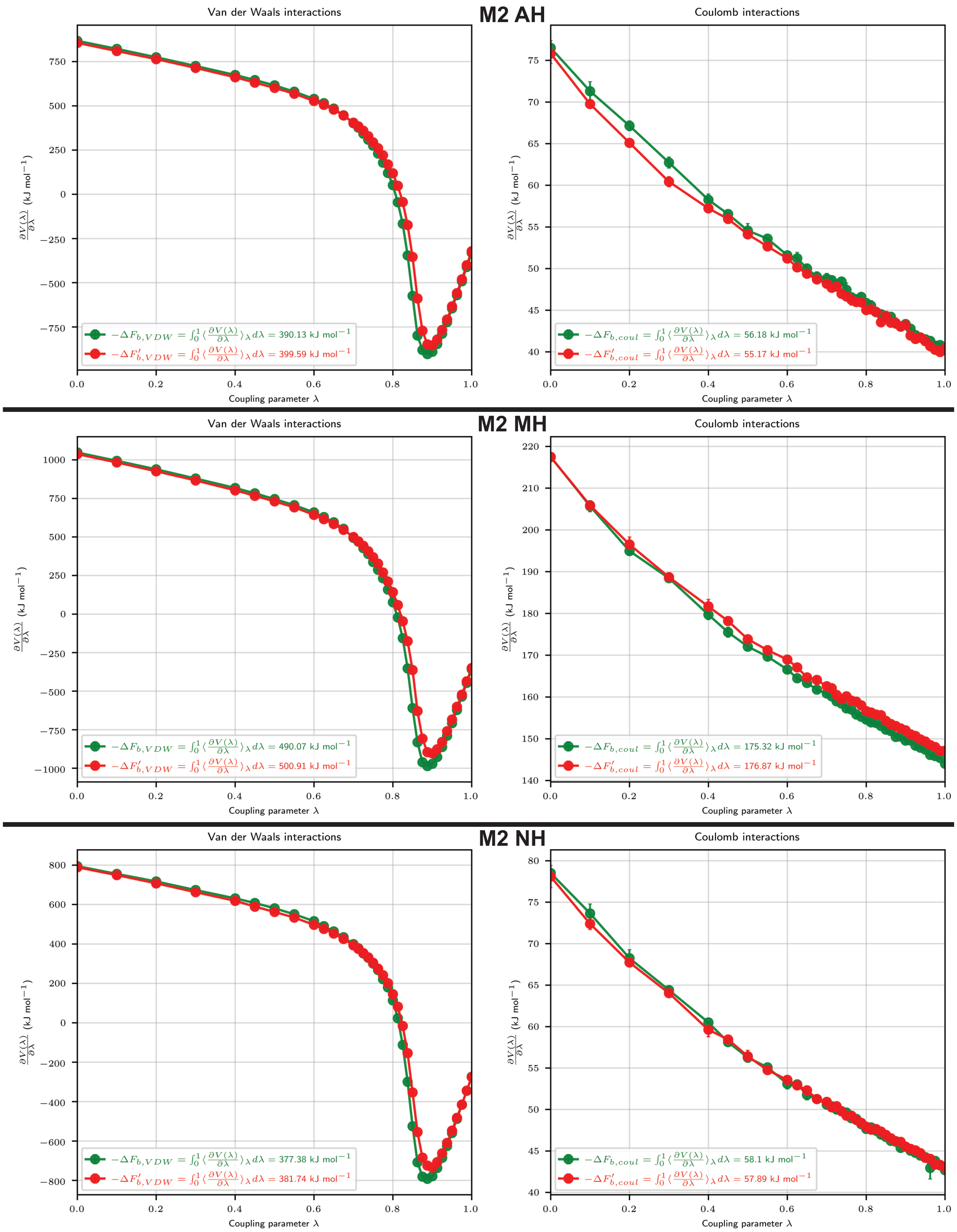

Figure SI2: The $\frac{\partial V}{\partial \lambda}$ -profiles for decoupling Van der Waals (left) and Coulomb (right) interactions for the tensionless system (green) and the tensed system (red). Integration of these curves yield free energy differences for the seperate interaction types (see legends). From these, we calculated $\Delta\Delta F_{sensing} = \Delta F'_b - \Delta F_b = (\Delta F'_{b,VDW} + \Delta F'_{b,coul}) - (\Delta F_{b,VDW} + \Delta F_{b,coul})$.

6 Density plots of peptide-membrane binding in different force-fields

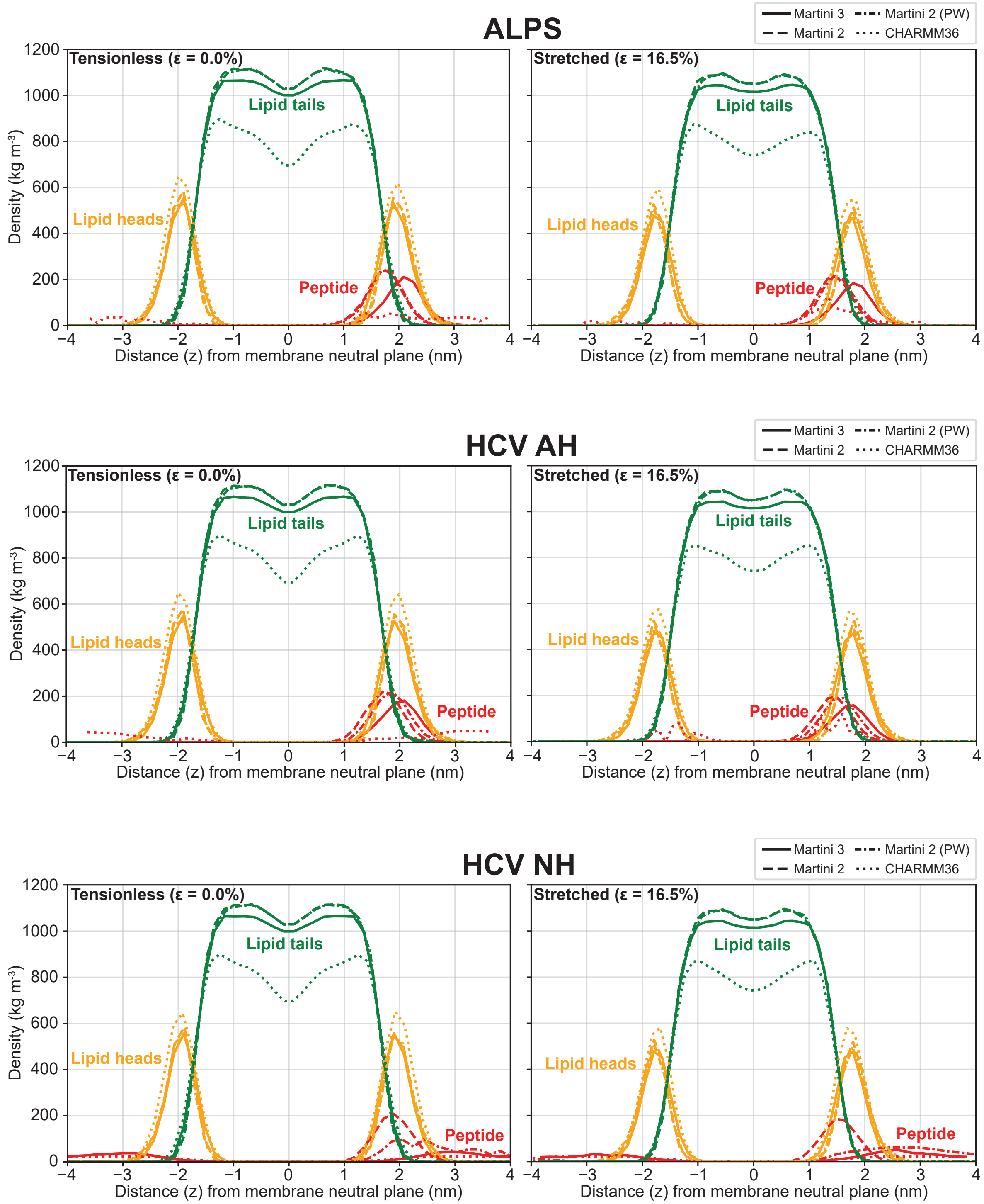

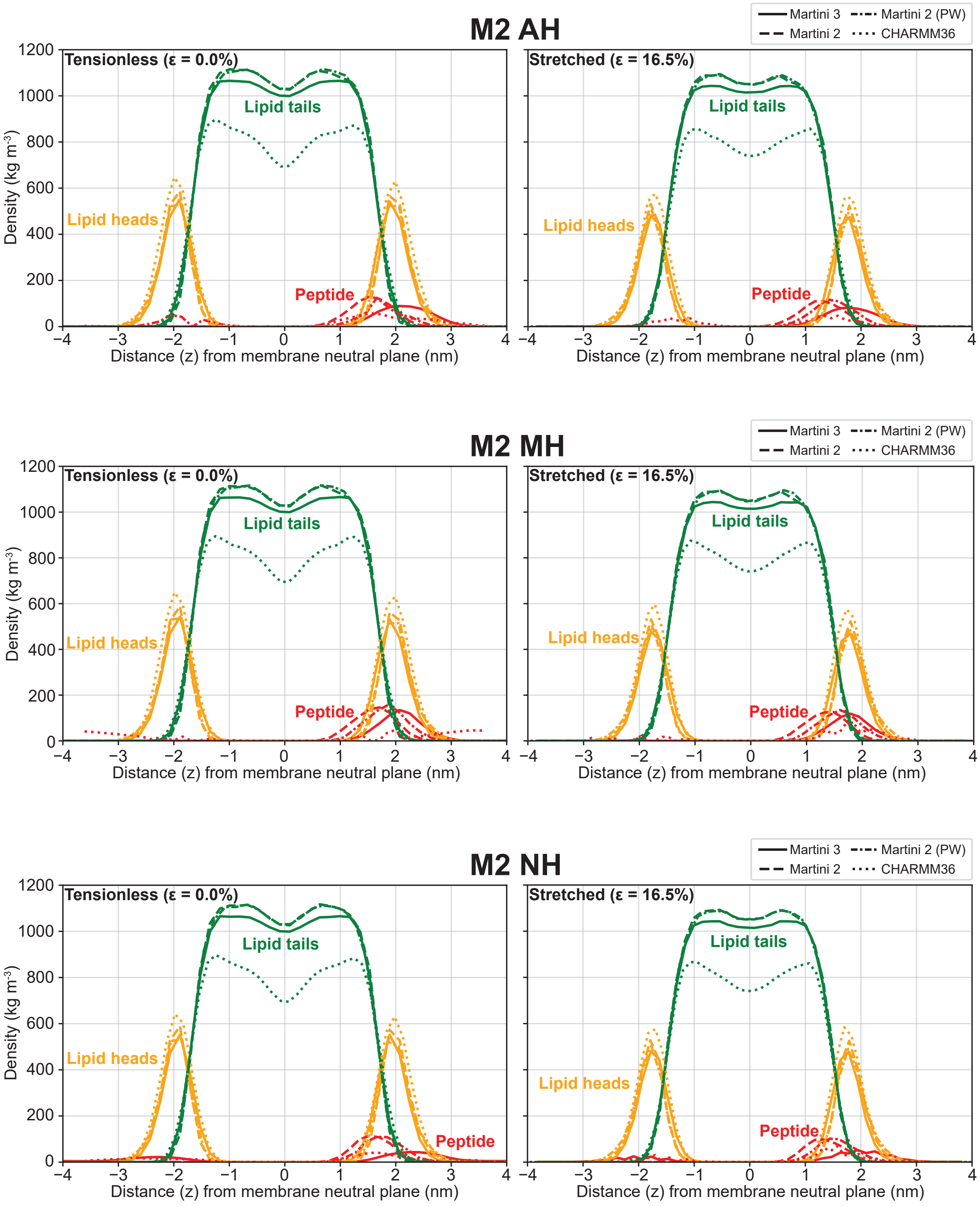

Figure SI3: Density plots calculated from MD trajectories (3 independent replicas) of peptides on tensionless (left) and stretched (right) POPC membranes, using different force-fields. Phosphocholine groups were taken as the 'lipid heads'. The alkyl tails and glycerol moieties are taken together as the 'lipid tails'. Solvent densities are not shown for clarity.

7 Insertion depths of peptide-membrane binding in different force-fields

Table SI3: For different force-fields, and in the tensionless (ϵ=0.0%) and stretched (ϵ=16.5%) states of the membrane, we report (left) the number of simulations in which the peptide is bound to the membrane for the majority of the trajectory (out of 3 replicas), (middle) the distances (z) from the bilayer neutral plane (in nm) for the lipid head groups (i.e. monolayer thickness), and the bound peptides, as calculated from the density plots (Fig. SI3), and (right) the relative position of the peptide with respect to the monolayer thickness, as percentages.

**Martini 3**	**Tensionless (ϵ=0.0%)**			**Stretched (ϵ=16.5%)**		
Monolayer thickness		1.903 ± 0.001			1.797 ± 0.002	
ALPS	3/3 bound	2.091 ± 0.002	110%	3/3 bound	1.798 ± 0.003	100%
HCV AH	3/3 bound	1.997 ± 0.002	105%	3/3 bound	1.704 ± 0.002	95%
HCV NH	0/3 bound	-	-	0/3 bound	-	-
M2 AH	3/3 bound	2.151 ± 0.006	113%	3/3 bound	1.824 ± 0.003	102%
M2 MH	3/3 bound	2.092 ± 0.003	110%	3/3 bound	1.776 ± 0.004	99%
M2 NH	0/3 bound	-	-	0/3 bound	-	-

**Martini 2**	**Tensionless (ϵ=0.0%)**			**Stretched (ϵ=16.5%)**		
Monolayer thickness		1.900 ± 0.002			1.795 ± 0.002	
ALPS	3/3 bound	1.740 ± 0.002	92%	3/3 bound	1.434 ± 0.001	80%
HCV AH	3/3 bound	1.722 ± 0.019	91%	3/3 bound	1.424 ± 0.002	79%
HCV NH	3/3 bound	1.897 ± 0.010	100%	3/3 bound	1.551 ± 0.008	86%
M2 AH	3/3 bound	1.555 ± 0.007	82%	3/3 bound	1.286 ± 0.004	72%
M2 MH	3/3 bound	1.616 ± 0.004	85%	3/3 bound	1.353 ± 0.004	75%
M2 NH	3/3 bound	1.590 ± 0.007	84%	3/3 bound	1.339 ± 0.004	75%

**Martini 2 (PW)**	**Tensionless (ϵ=0.0%)**			**Stretched (ϵ=16.5%)**		
Monolayer thickness		1.864 ± 0.003			1.778 ± 0.000	
ALPS	3/3 bound	1.756 ± 0.015	94%	3/3 bound	1.485 ± 0.001	84%
HCV AH	3/3 bound	1.832 ± 0.013	98%	3/3 bound	1.543 ± 0.002	86%
HCV NH	0/3 bound	-	-	0/3 bound	-	-
M2 AH	3/3 bound	1.794 ± 0.007	96%	3/3 bound	1.492 ± 0.002	84%
M2 MH	3/3 bound	1.829 ± 0.007	98%	3/3 bound	1.580 ± 0.007	89%
M2 NH	3/3 bound	1.780 ± 0.058	95%	3/3 bound	1.543 ± 0.006	87%

**CHARMM36**	**Tensionless (ϵ=0.0%)**			**Stretched (ϵ=16.5%)**		
Monolayer thickness		1.989 ± 0.003			1.717 ± 0.001	
ALPS	0/3 bound	-	-	2/3 bound	1.425 ± 0.008	83%
HCV AH	0/3 bound	-	-	3/3 bound	1.451 ± 0.022	85%
HCV NH	0/3 bound	-	-	0/3 bound	-	-
M2 AH	3/3 bound	1.796 ± 0.099	90%	3/3 bound	1.429 ± 0.084	83%
M2 MH	2/3 bound	1.836 ± 0.086	92%	3/3 bound	1.526 ± 0.384	89%
M2 NH	2/3 bound	1.665 ± 0.145	84%	3/3 bound	1.511 ± 0.036	88%

8 Effect of secondary and tertiary structure on CHAOS parameter

As the Martini CG force-field does not model protein folding dynamics, secondary structure is imposed through stringent (dihedral) angles between the backbone beads. As the peptides studied here – and most other known membrane surface peptides – are α-helical upon binding to the membrane, we applied this enforced folding to all peptides examined in this work. To assess to what extent this influences lipid packing sensing and tension induction, we calculated CHAOS values for the six peptides we studied throughout this paper with enforced α-helicity and without it (random coil), see Fig. SI4A.

For the six peptides tested here, the helix-to-coil transition least affected the M2 peptides, which are intrinsically less flexible than ALPS and the HCV peptides due to their shorter length. Most prominently, the CHAOS value for the coil configuration of HCV NH increased with respect to the helical fold. This increase can be attributed to the gained flexibility allowing for repositioning and insertion of the four tryptophan residues that is lost when the peptide is forced to fold into an α-helix. Finally, we conclude that for all active peptides (ALPS, HCV AH, M2 AH, and M2 MH), most of the sensing and tension inducing activity (i.e. CHAOS > 0.4) is retained when the peptides lose their fixed α-helical secondary structure.

Helix-turn-helix tertiary structure of HCV AH

As a special case, HCV AH was originally assumed to fold into a straight α-helix[3,4] (as also predicted by AlphaFold2,[5] Fig. SI4B), whereas a recent study used a helix-turn-helix (HTH) tertiary structure for HCV AH[1] as a starting point for their MD simulations (as predicted by PEP-FOLD,[6] Fig. SI4C). This already indicates that the folding of HCV AH is not trivially defined, likely very dynamic and dependent on the environment (in aqueous solvent versus membrane-bound). We simulated the HTH conformation and found that a CHAOS

parameter of 0.672 ± 0.032 nm^2, which falls between the helix and coil values (Fig. SI4A).

To illustrate the dynamics of HCV AH in the presence of a (tensionless) membrane, we analyzed the end-to-end distance between the the peptide's termini (S1 and L27 backbone/C_α atoms) in our atomistic and CG trajectories, starting from an α-helix (Fig. SI4D). In line with the known limitations of the Martini model considering protein folding, we observed that the CG model retains the initial α-helical configuration (long end-to-end distance). In contrast, the atomistic model dynamically transitions to a partly unfolded HTH conformation with a shorter end-to-end distance (Fig. SI4D), and low affinity for the tensionless membrane (consistent with previous findings[1]). It is important to note that our current data do not provide fully conclusive answers in this regard, since both peptide folding and membrane binding are processes that have timescales of multiple microseconds and therefore usually require enhanced sampling methods to capture properly, which is beyond the scope of the current work.

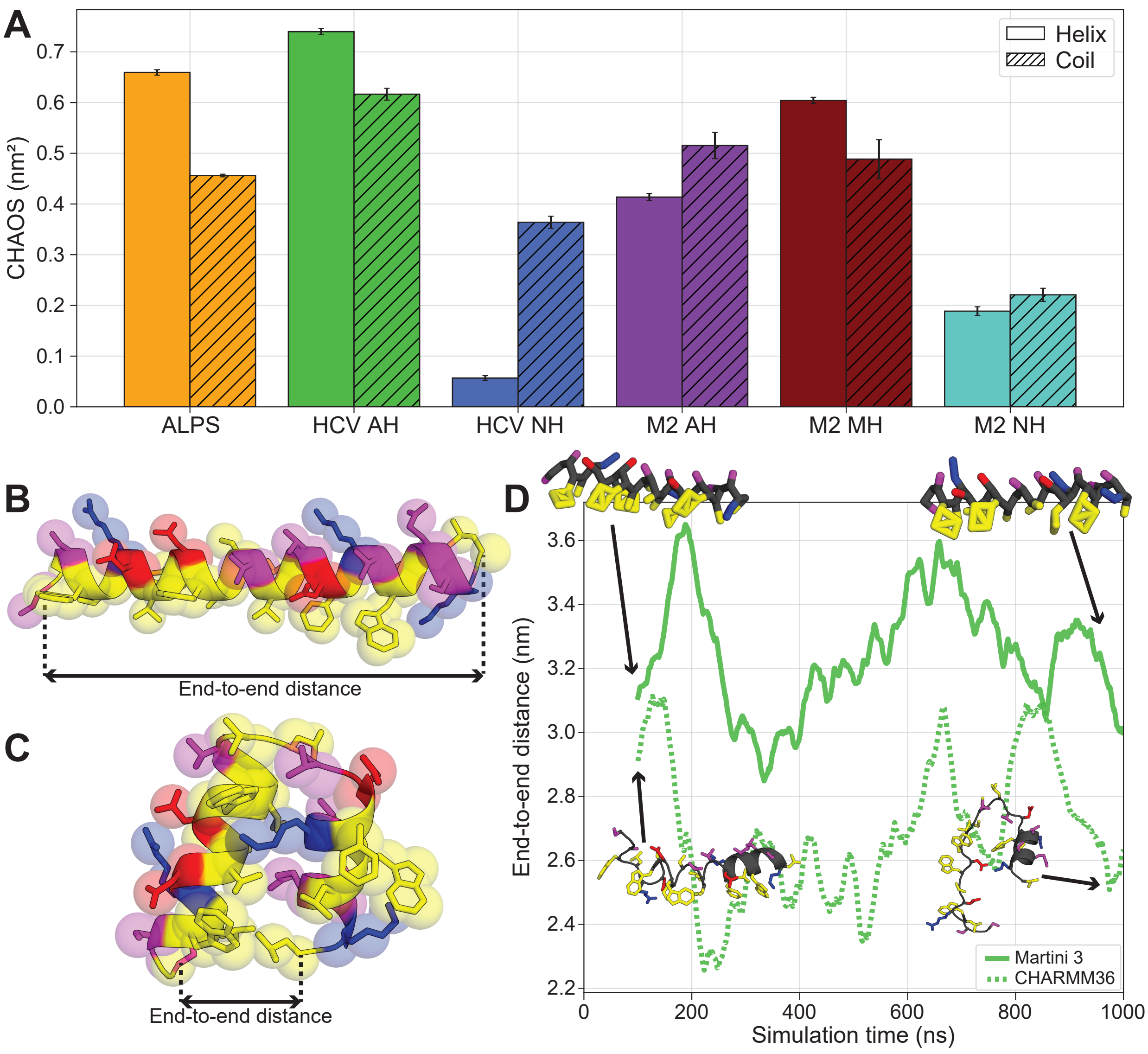

Figure SI4: A) CHAOS parameter values for helix and coil configurations of our six peptides of interest (averages and standard deviations for three independent replica runs). B) Predicted atomistic structures for HCV AH by AlphaFold2.[5] C) The HTH-tertiary structure, as predicted by PEP-FOLD.[6] D) The end-to-end distance between the atomistic and CG peptide termini (100 ns moving average) in the presence of a tensionless membrane. Insets: atomistic and CG snapshots of the peptide structures (lipids and solvent not shown for clarity).

References

(1) Chng, C.-P.; Cho, N.-J.; Hsia, K. J.; Huang, C. Role of Membrane Stretch in Adsorption of Antiviral Peptides onto Lipid Membranes and Membrane Pore Formation. *Langmuir* **2021**,

(2) Gautier, R.; Douguet, D.; Antonny, B.; Drin, G. HELIQUEST: a web server to screen sequences with specific α-helical properties. *Bioinformatics* **2008**, *24*, 2101–2102.

(3) Cho, N.-J.; Dvory-Sobol, H.; Xiong, A.; Cho, S.-J.; Frank, C. W.; Glenn, J. S. Mechanism of an Amphipathic α-Helical Peptide's Antiviral Activity Involves Size-Dependent Virus Particle Lysis. *ACS Chem. Biol.* **2009**, *4*, 1061–1067.

(4) Park, S.; Jackman, J. A.; Cho, N.-J. Comparing the Membrane-Interaction Profiles of Two Antiviral Peptides: Insights into Structure–Function Relationship. *Langmuir* **2019**, *35*, 9934–9943.

(5) Jumper, J.; Evans, R.; Pritzel, A.; Green, T.; Figurnov, M.; Ronneberger, O.; Tunyasuvunakool, K.; Bates, R.; Žídek, A.; Potapenko, A.; Bridgland, A.; Meyer, C.; Kohl, S. A. A.; Ballard, A. J.; Cowie, A.; Romera-Paredes, B.; Nikolov, S.; Jain, R.; Adler, J.; Back, T.; Petersen, S.; Reiman, D.; Clancy, E.; Zielinski, M.; Steinegger, M.; Pacholska, M.; Berghammer, T.; Bodenstein, S.; Silver, D.; Vinyals, O.; Senior, A. W.; Kavukcuoglu, K.; Kohli, P.; Hassabis, D. Highly accurate protein structure prediction with AlphaFold. *Nature* **2021**, *596*, 583–589.

(6) Lamiable, A.; Thévenet, P.; Rey, J.; Vavrusa, M.; Derreumaux, P.; Tufféry, P. PEP-FOLD3: fasterde novostructure prediction for linear peptides in solution and in complex. *Nucleic Acids Res.* **2016**, *44*, W449–W454.
